# Supplementary material for: Toward grouped-reservoir computing: organic neuromorphic vertical transistor with distributed reservoir states for efficient recognition and prediction
Source: Nat Commun. 2024 Jan 25;15:740. doi: 10.1038/s41467-024-44942-8 (PMC10810880; doi:10.1038/s41467-024-44942-8)
Supplement: Supplementary file 1 — Supplementary Information [file 41467_2024_44942_MOESM1_ESM.pdf]

Toward grouped-reservoir computing: organic neuromorphic vertical transistor with distributed reservoir states for efficient recognition and prediction

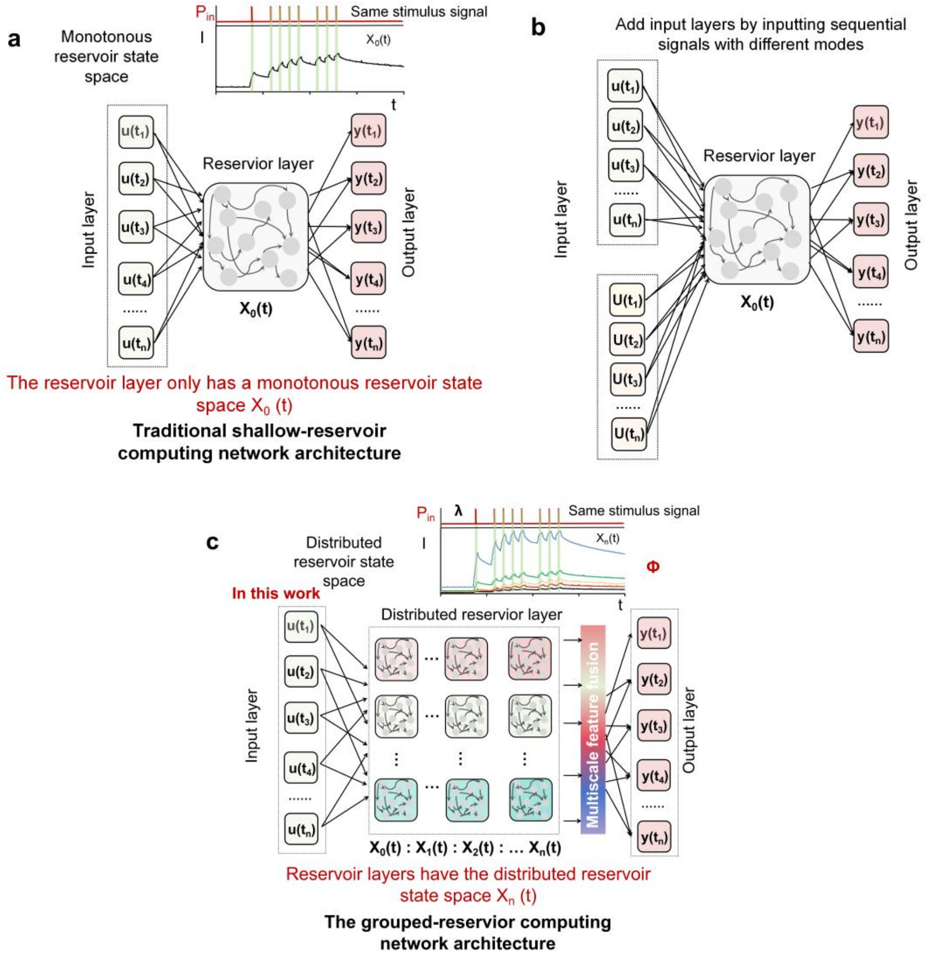

Supplementary Information Fig. S1. | Different architectures of reservoir computing. a The network architecture for traditional shallow-reservoir computing has a single reservoir state space. **b** By increasing the number of input layers, the timescale range of the reservoir can be enhanced, but the reservoir state space remains monotonic. **c** This work proposes a grouped-reservoir computing based on organic vertical neuromorphic transistors, which consists of distributed reservoir with state spaces having different spatiotemporal characteristics.

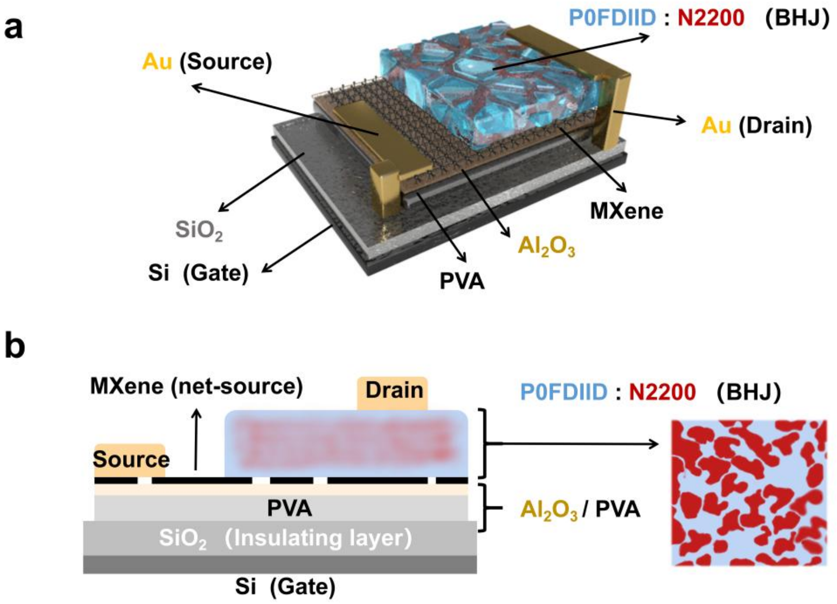

Supplementary Information Fig. S2 | Schematic diagram of the structure of VOFET-DR.

a The three-dimensional device structure diagram of VOFET-DR is used in the main manuscript to clearly show the architecture of the device. **b** Schematic diagram of the two-dimensional cross-sectional structure of VOFET-DR. Si is the gate electrode. SiO_2 is the insulating layer. Al_2O_3/PVA is used as the interface passivation layer to reduce the off current of the device. MXene is used as the network source of the vertical transistor. The mixed organic semiconductor thin film of P0FDIID: N2200 is used as the channel. The Au on the left is used as the probe for the source to be connected to the tester during testing. The Au on the right is used as the drain.

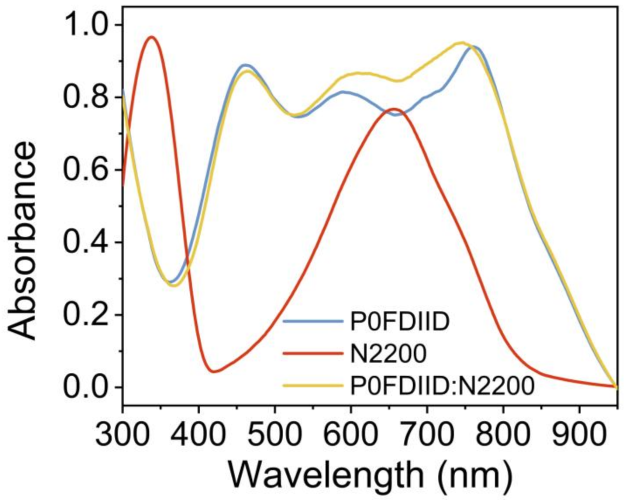

Supplementary Information Fig. S3 | Absorption spectrum of active layer materials.

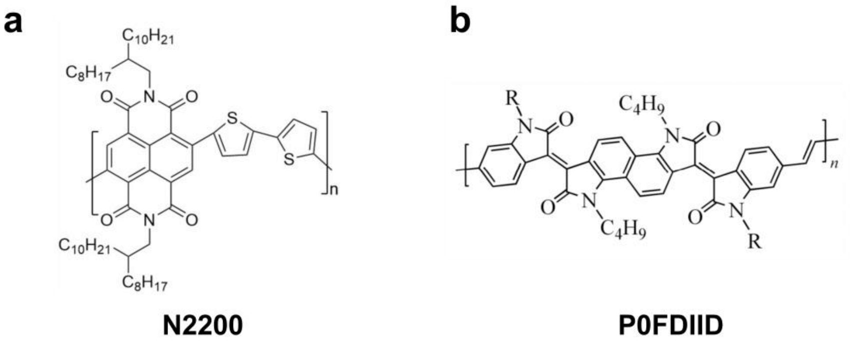

Supplementary Information Fig. S4 | Chemical molecular structure of active layer materials.

a and **b** shows the molecular structures of N2200 and P0FDIID, respectively.

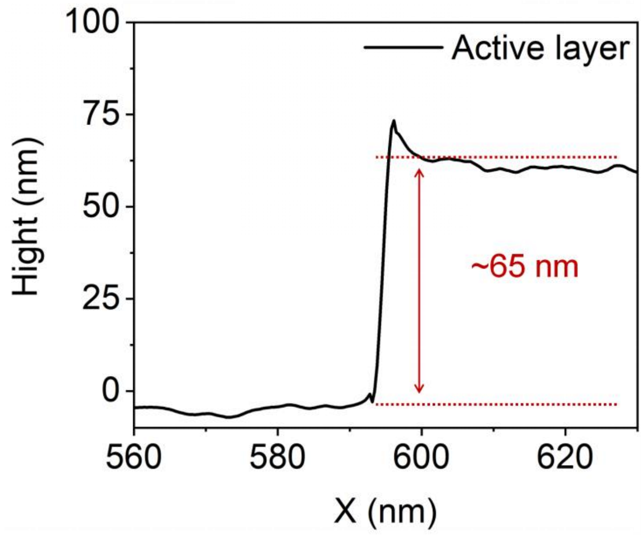

Supplementary Information Fig. S5 | The thickness of the active layer is about 65 nm.

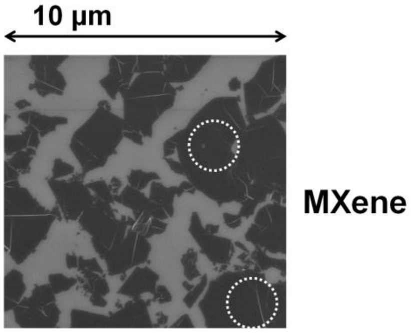

Supplementary Information Fig. S6 | SEM image of MXene film with 3 mg /ml concentration.

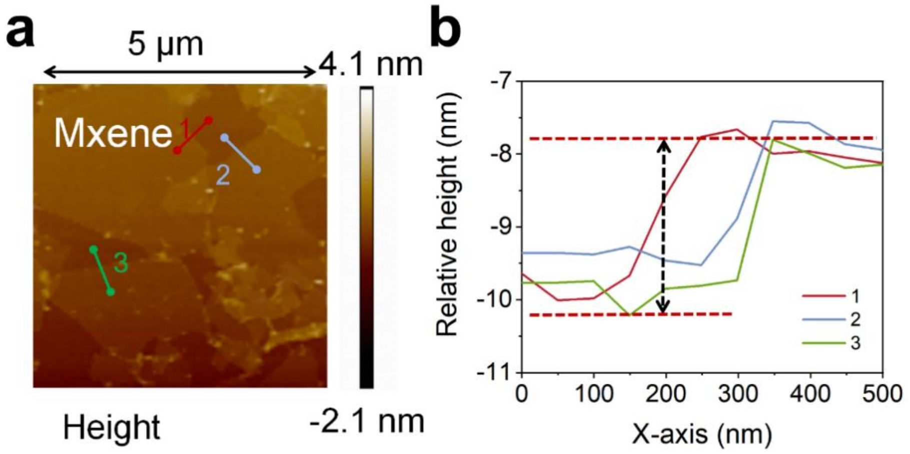

Supplementary Information Fig. S7 | Height image of MXene. a Height map of MXene mesh source electrode tested by atomic force microscopy. **b** Thickness of film at positions 1, 2 and 3.

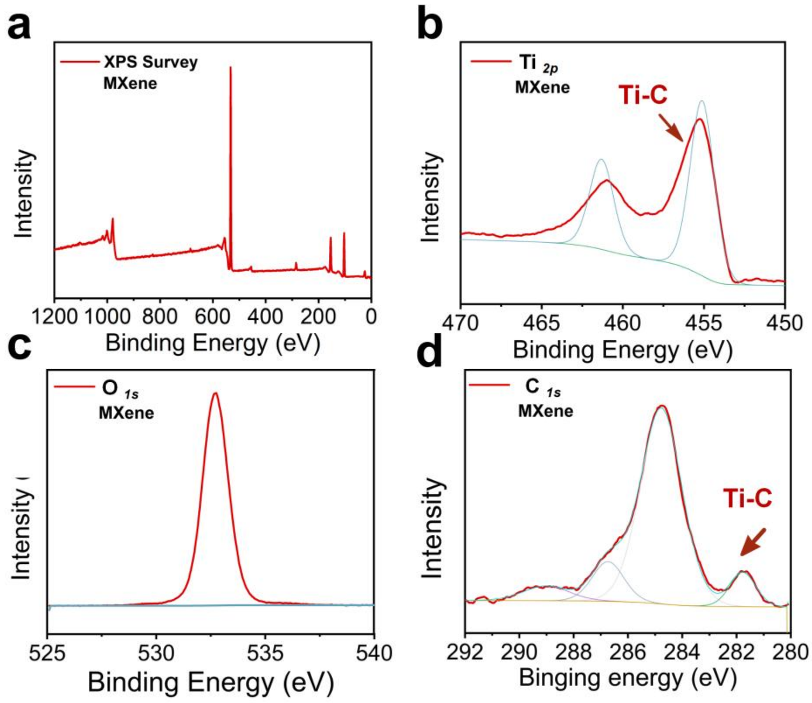

Supplementary Information Fig. S8 | XPS spectra of the surface of MXene films. **a** The survey results on the surface of MXene films. Scanning results of **b** Ti $_{2p}$, **c** O $_{1s}$ and **d** C $_{1s}$ on the surface of MXene films.

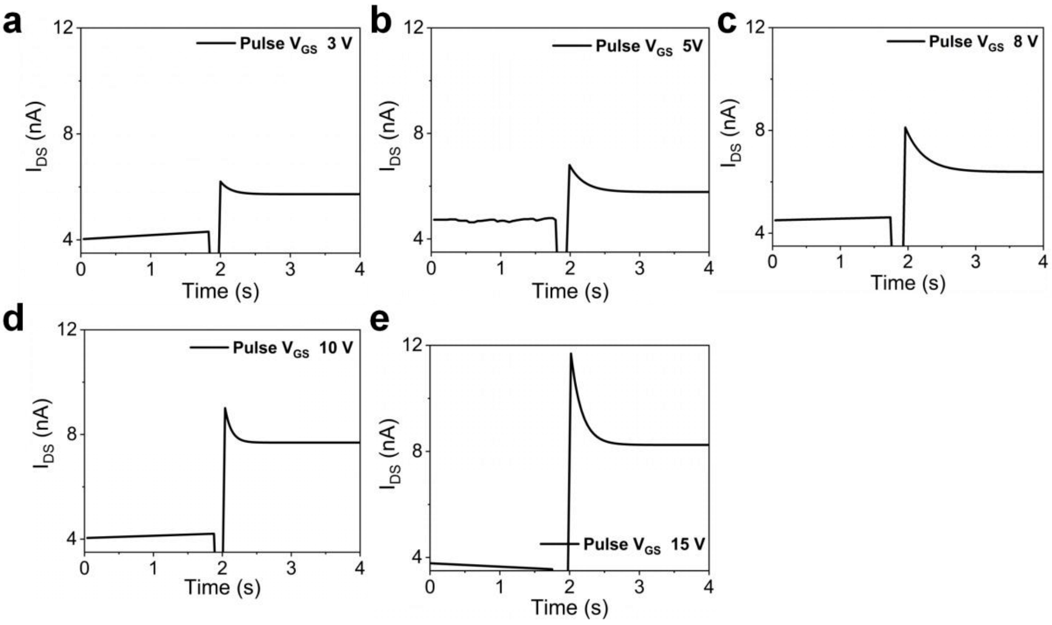

Supplementary Information Fig. S9 | The I_{DS}-t curve of the device after being input to the electrical pulse. V_{GS} electrical pulses of **a** 3 V, **b** 5 V, **c** 8 V, **d** 10 V, **e** 15 V are applied separately.

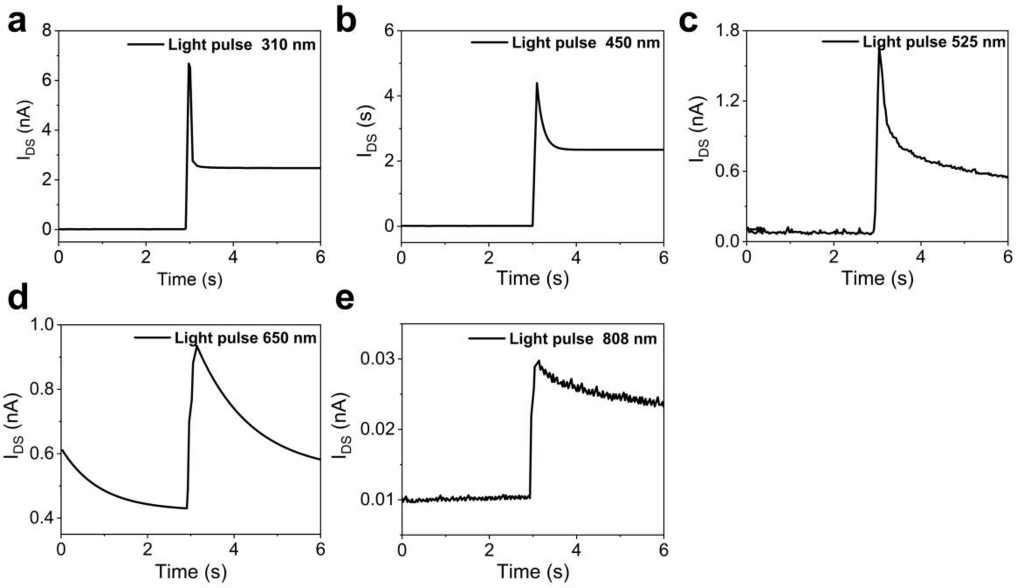

Supplementary Information Fig. S10 | The I_{DS}-t curve of the device after being input to the light pulse. Light pulses of **a** 310 nm, **b** 450 nm, **c** 525, **d** 650 nm, **e** 808 nm are individually applied.

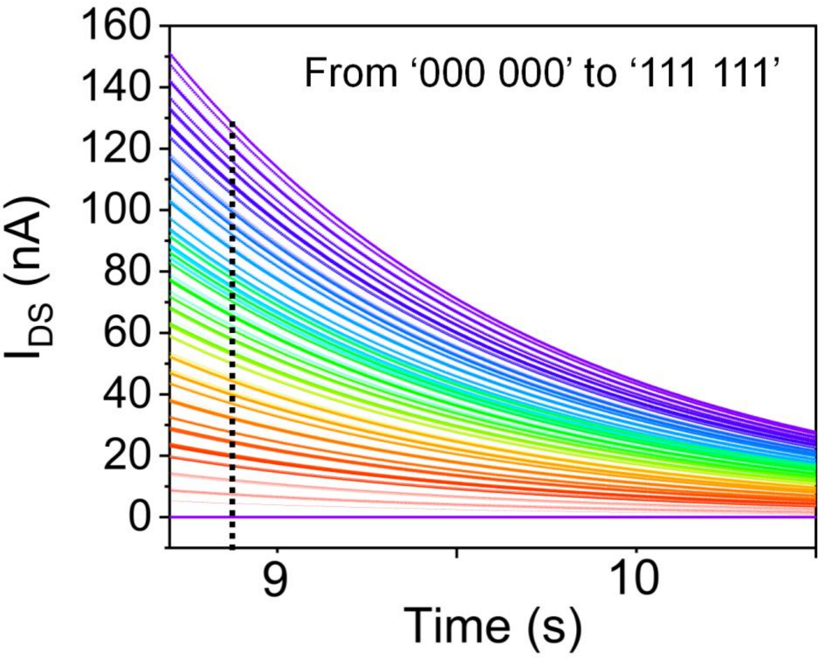

Supplementary Information Fig. S11 | Reservoir state x (t_6) of the device after 64 different sequence light signals are input respectively.

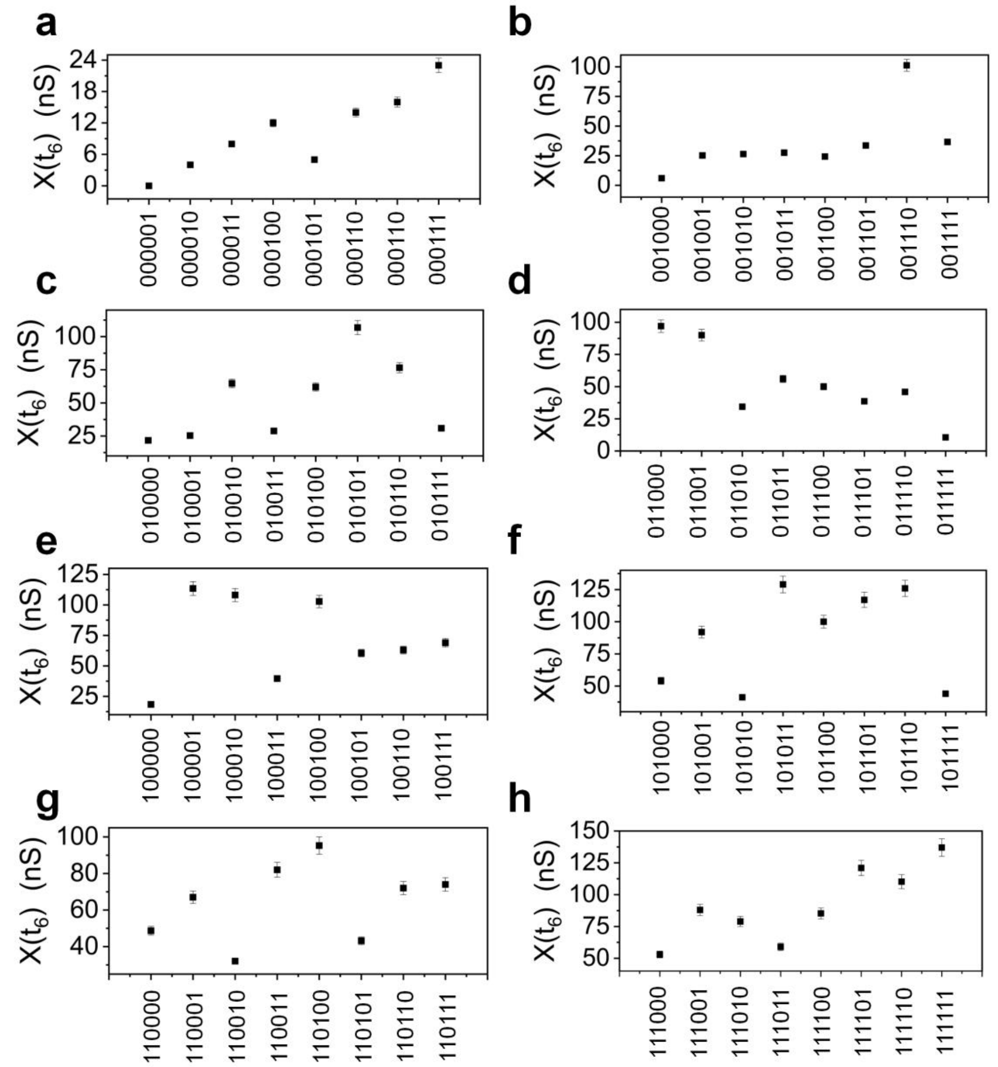

Supplementary Information Fig. S12 | Error of reservoir state x (t_6) of the device after 64 different sequence light signals are input respectively. The error bar range is derived from the maximum and minimum values after 5 samples of experimental data. a Eight sequences of light signals from 000000 to 000111. **b** From 001000 to 001111. **c** From 010000 to 010111. **d** From 011000 to 011111. **e** From 100000 to 100111. **f** From 101000 to 101111. **g** From 110000 to 110111. **h** From 111000 to 111111.

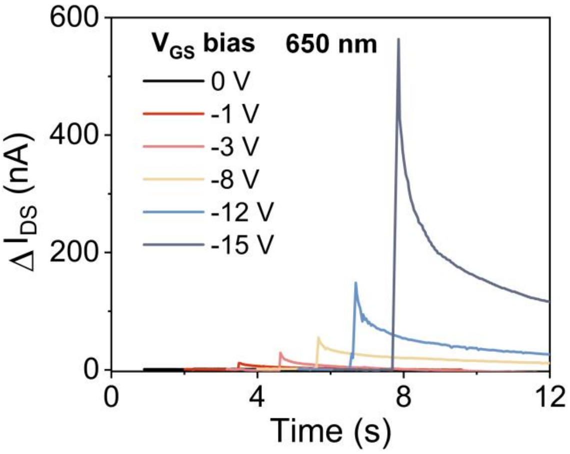

Supplementary Information Fig. S13 | I_{DS} - t curves after a single light pulse of 650 nm is input to VOFET-DR with different gate biases (V_{DS} = -1V).

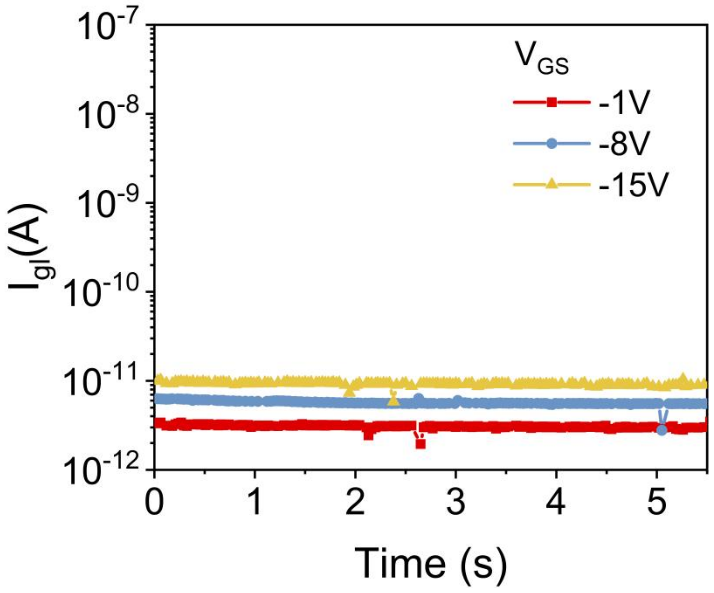

Supplementary Information Fig. S14 | Gate leakage current of the VOFET-DR.

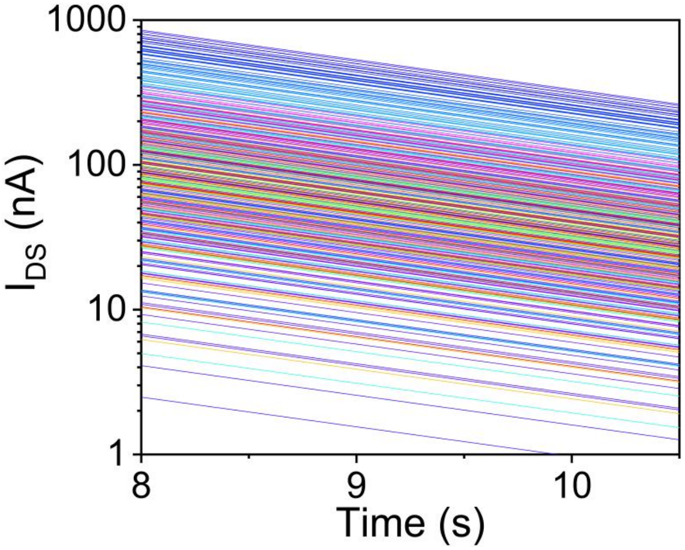

Supplementary Information Fig. S15 | Different bias modulation. Under the conditions of different bias V_{GS}, (0V, -1V, -3V, -8V, -12V, -15V, V_{DS} = -1V), the device is input from 64 kinds of optical pulse sequence signals ranging from '000000' to '111111', which produces 384 reservoir states.

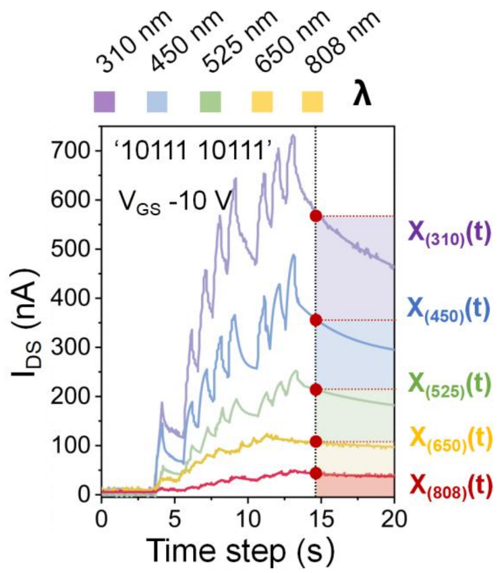

Supplementary Information Fig. S16 | The effect of wavelength on physical node mapping sequence signals.

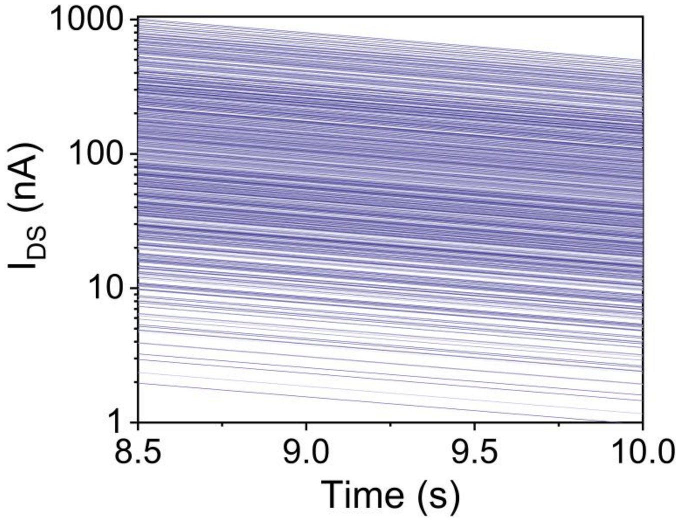

Supplementary Information Fig. S17 | Optical and electrical coupling modulation. Through the optical pulse input signal in three bands of ultraviolet (310 nm), visible (650nm) and near-infrared (808 nm) light, ranging from '000000' to '111111', combined with different bias V_{GS} (0V, -1V, -3V, -8V, -12V, -15), the device can obtain 1152 reservoir states.

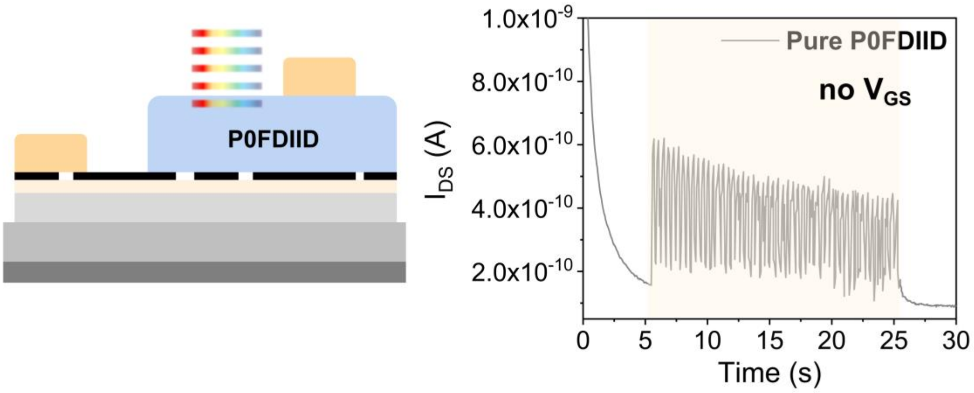

Supplementary Information Fig. S18 | The I_{DS}-t obtained by continuously inputting light pulses to devices without N2200.

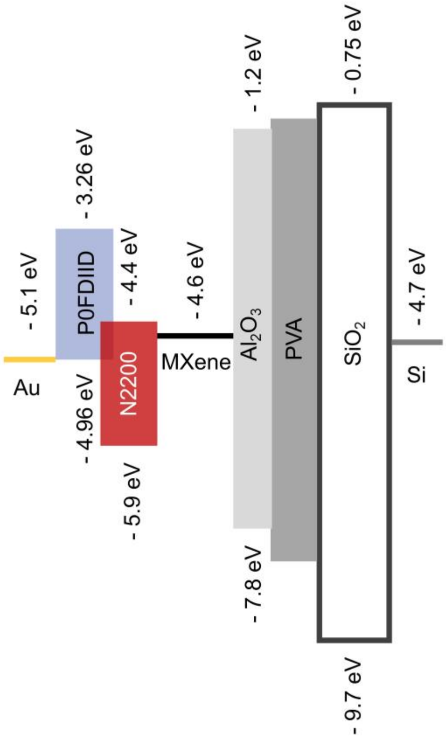

Supplementary Information Fig. S19 | The energy band structure of the material.

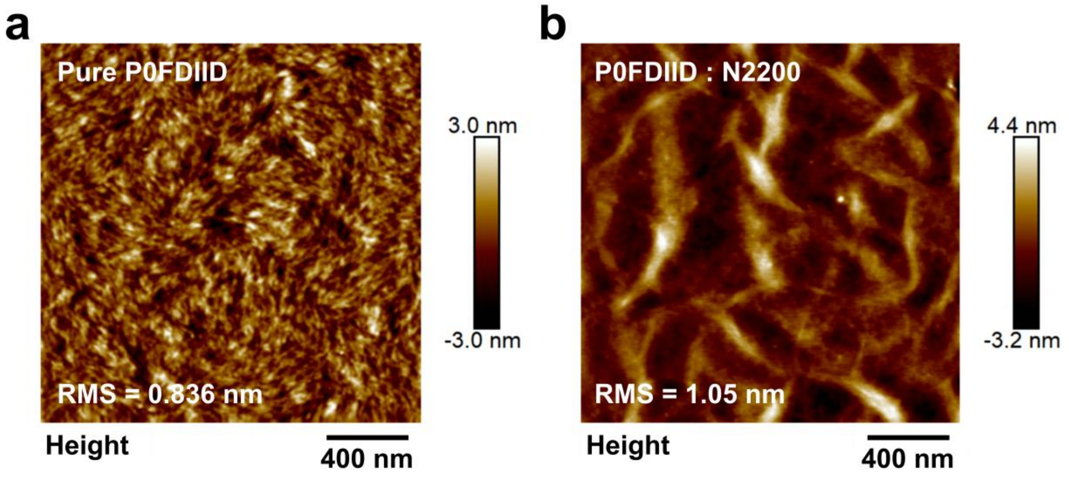

Supplementary Information Fig. S20 | Morphology of mixed films and pure P0FDIID films tested by atomic force microscopy.

a Morphology of pure P0FDIID film with a roughness of 0.836 nm. **b** Morphology of P0FDIID and N2200 bulk heterojunction film with a roughness of 1.05 nm.

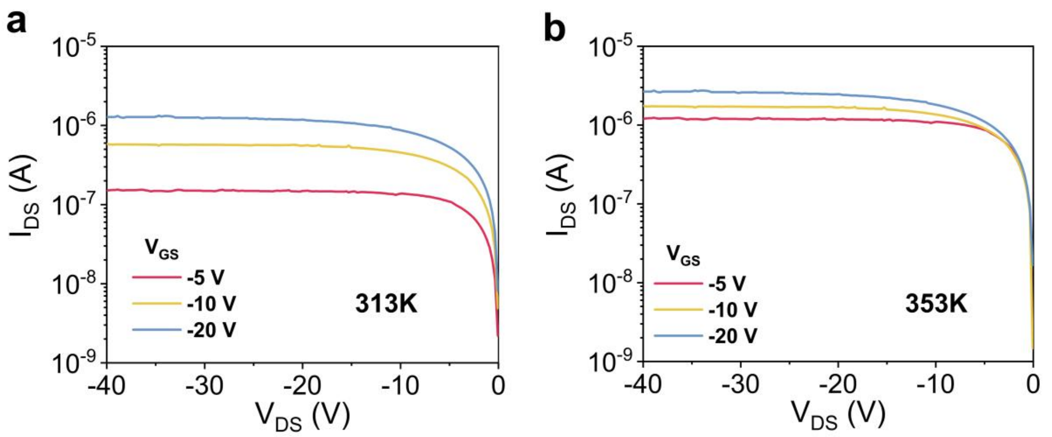

Supplementary Information Fig. S21 | The temperature-dependent output characteristics of VOFET-DR.

a The output characteristic of VOFET-DR under 313K and **b** 353 K.

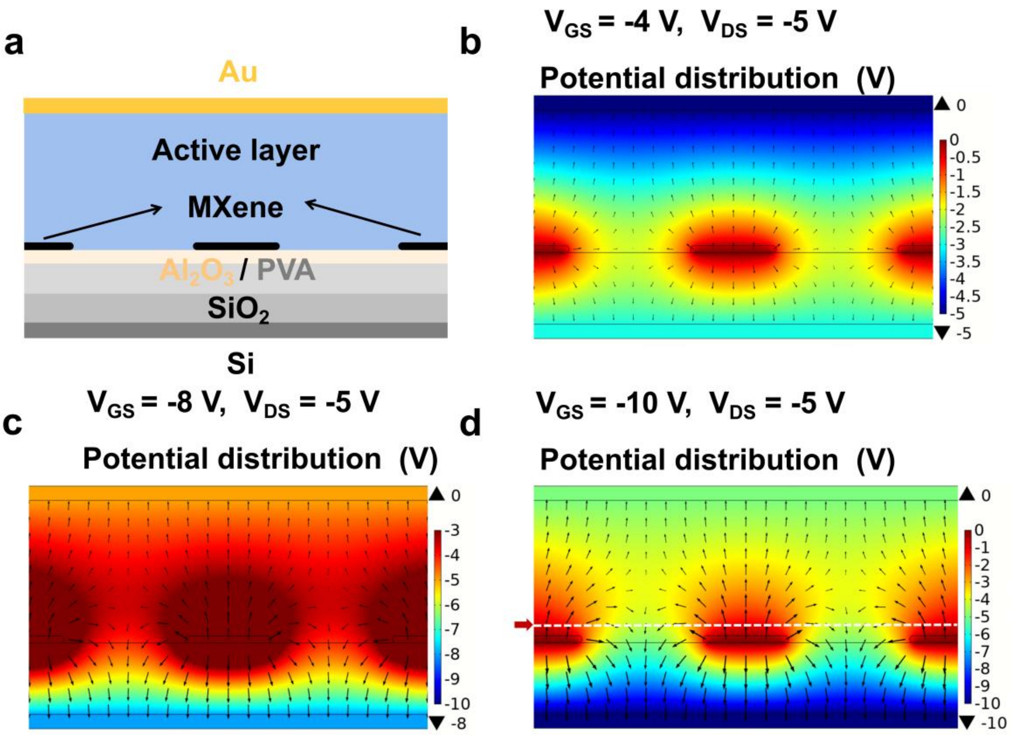

Supplementary Information Fig. S22 | Potential distribution of the device for V_{GS} <0.

a Schematic diagram of 2D cross-section simulation calculation of the device. The potential distribution inside the device at **b** V_{GS} = -4 V, **c** V_{GS} = -8 V, and **d** V_{GS} = -10 V, respectively.

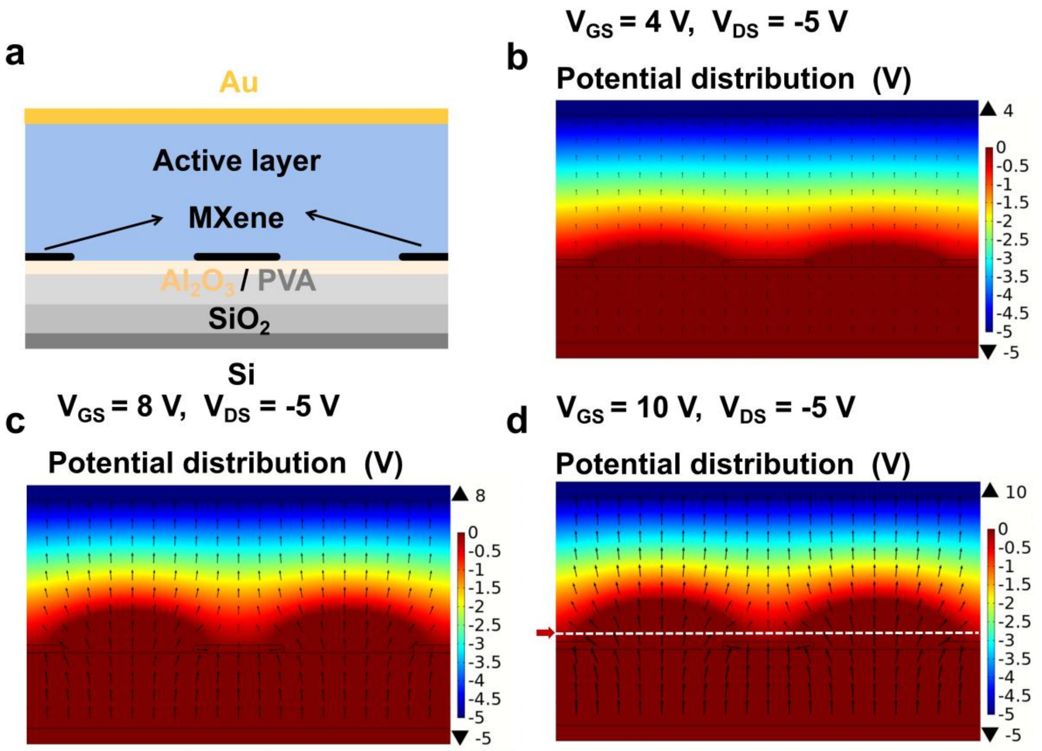

Supplementary Information Fig. S23 | Potential distribution of the device for V_{GS} >0.

a Schematic diagram of 2D cross-section simulation calculation of the device. The potential distribution inside the device at **b** V_{GS} = +4 V, **c** V_{GS} = +8 V, and **d** V_{GS} = +10 V, respectively.

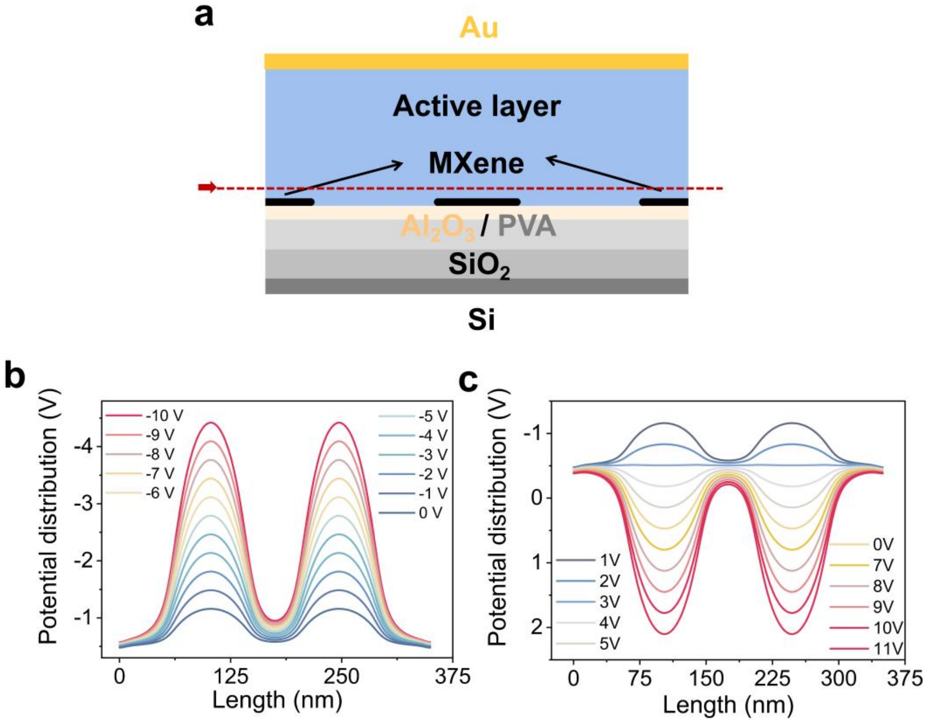

Supplementary Information Fig. S24 | Potential distribution of the device.

a Schematic diagram of 2D cross-section simulation calculation of the device.

b The potential distribution extracted from the dashed line in **a** evolves for $V_{GS} < 0$.

c The potential distribution extracted from the dashed line in **a** evolves for $V_{GS} > 0$.

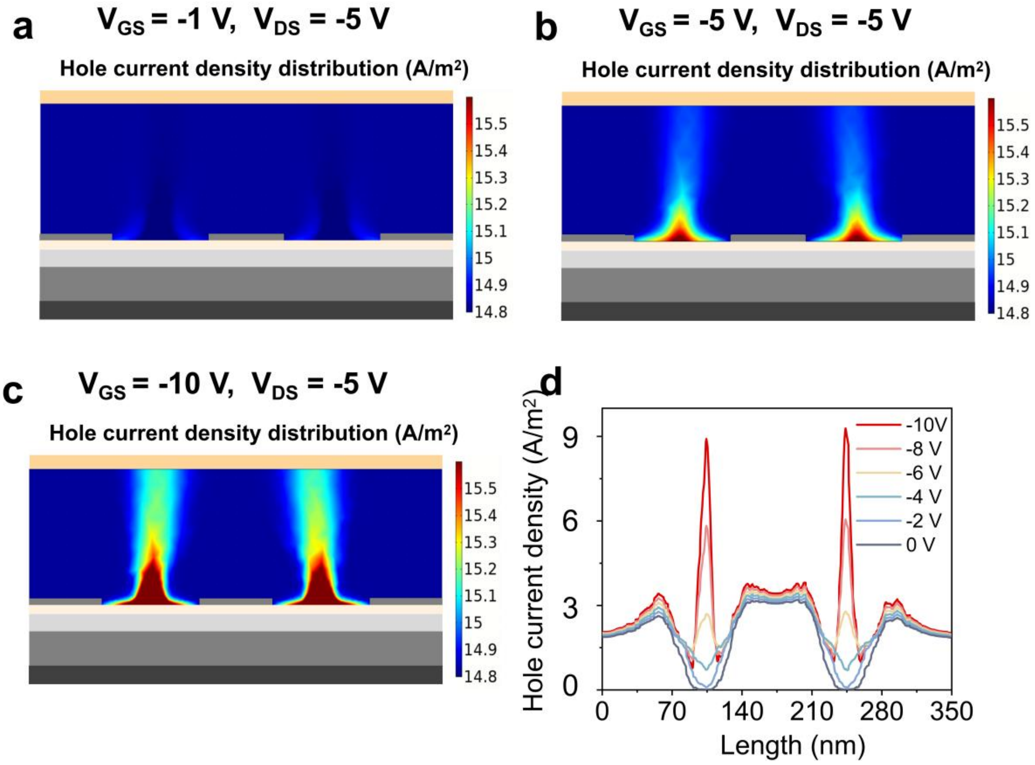

Supplementary Information Fig. S25 | Current density distribution of the device for V_{GS} >0.

The current density distribution inside the device at **a** V_{GS} = -1 V, **b** V_{GS} = -5 V, and **c** V_{GS} = -10 V, respectively.

d The current density distribution extracted from the dashed line in **Fig. S22a** evolves for $V_{GS} < 0$.

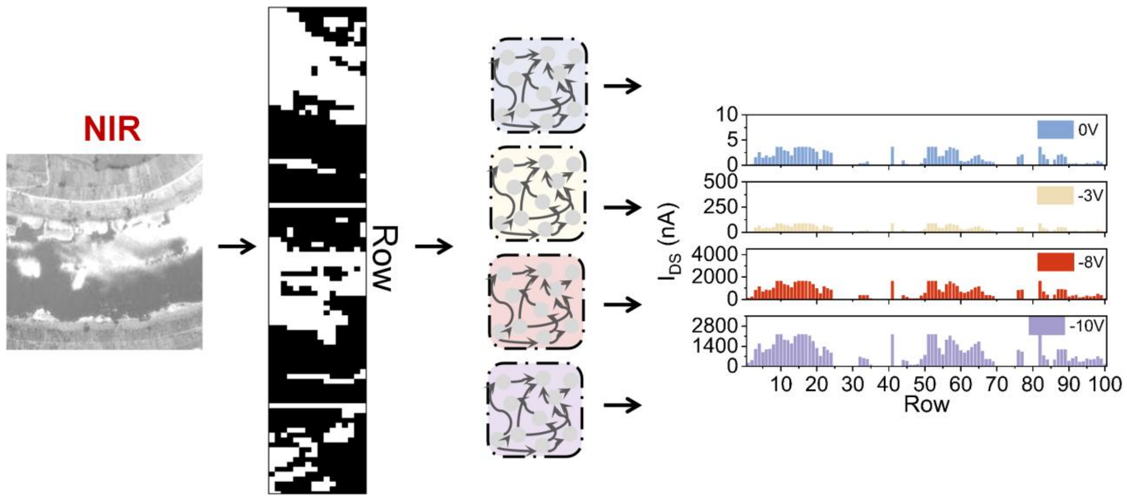

Supplementary Information Fig. S26 | Mapping sequence signals. Each sub-reservoir includes 100 VOFET-DR physical nodes, and generates 100 feature outputs for feature fusion and training of the output layer

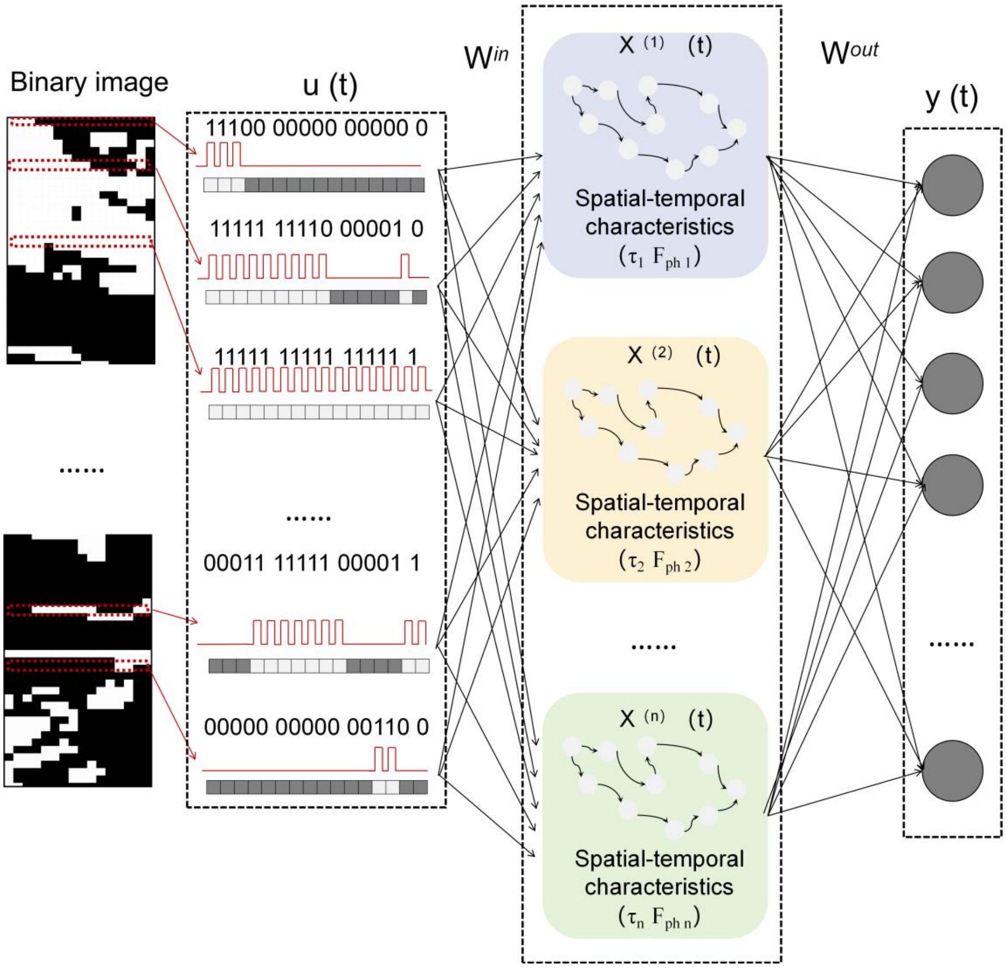

Supplementary Information Fig. S27 | Schematic diagram of grouped reservoir computing network proposed in this work. The sub-reservoirs with different spatiotemporal characteristics in the reservoir are composed of VOFET-DR. By applying different gate biases to VOFET-DR, the device has rich carrier dynamics under the coupling action of field effect and persistent photoconductivity effect. Therefore, the sequence signal u (t) can be mapped into different reservoir state spaces.

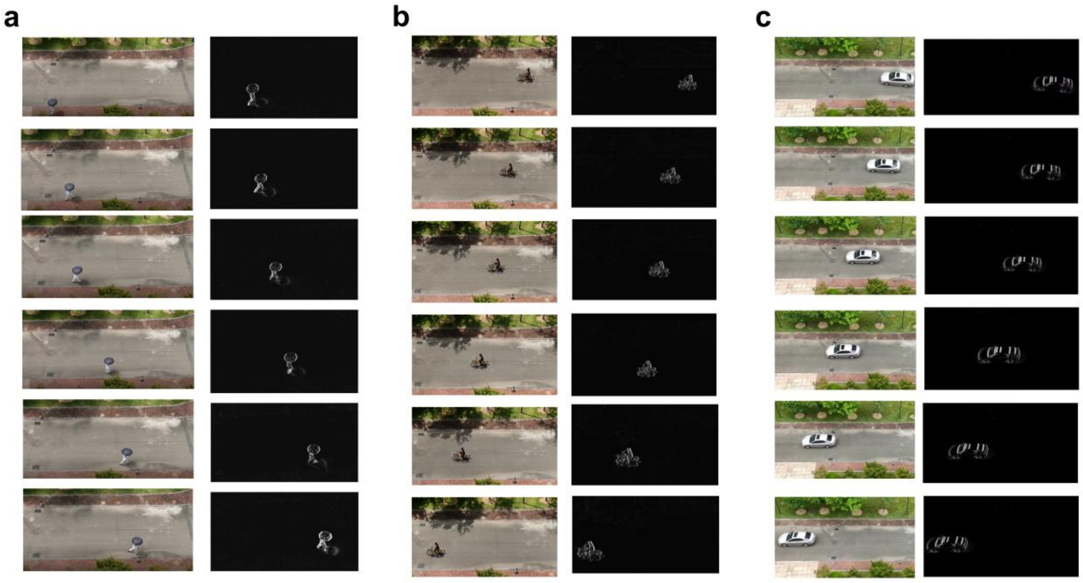

Supplementary Information Fig. S28 | The spatial frame information of different traffic elements. The spatiotemporal frame information of **a** pedestrians, **b** bicycles, and **c** cars.

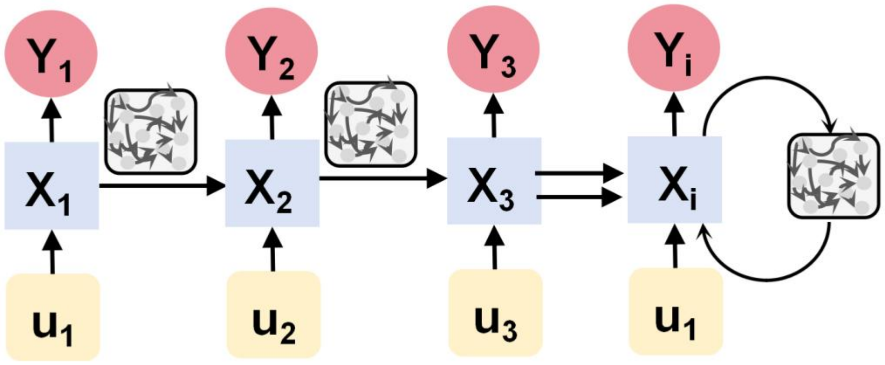

Supplementary Information Fig. S29 | Structure of the VOFET-DR system for motion prediction. u_1 indicates the first frame for prediction, X_1 is the reservoir state at the current moment of the autoencoder network, and Y_i is the predicted output frame.

Device structure	Active layer	Modal diversity of signal	Range ratio of F_E (Max/Min)	Range ratio of F_{ph} (nA/mW cm^{-2}) (Max/Min)	Range ratio of τ (s) (Max/Min)	Reservoir states	RC - architecture	Work
Memristor	WO_x	Electricity	1 (0.19/0.19)	N/A	1 (0.05/0.05)	10	Shallow -RC	1
Memristor	TiO_x / TaO_y	Electricity	1 (5.07/5.07)	N/A	1 (0.0004/0.0004)	N/A	Shallow-RC	2
Memristor	TaO_x / HfO_y	Light (208nm)	N/A	1 ($6\times10^4/6\times10^4$)	1 (0.3/0.3)	32	Shallow-RC	3
Memristor	α -In_2Se_3	Electicity or Light (532 nm)	2.17 (0.13/0.06)	4.8 (3.75/0.78)	1000 (10/0.1)	32	Shallow-RC	4
Memristor	BFO/SRO	Electicity	1 (0.16/0.16)	N/A	1 (0.012/0.012)	18	Shallow-RC	5
Memristor	ZnO	Light (320nm-400nm)	N/A	1 (3.07/3.07)	1 (0.18/0.18)	300	Shallow-RC	6
Planar field effect transistor	p-NDI	Light (Visual)	N/A	5 (50/10)	210 (2.1/0.01)	32	Shallow-RC	7
Vertical field effect transistor	P0FDIID: N2200	Electicity or Light (310nm - 808nm)	2.4 （72/30）	650 (5.2×10^4/80)	2640 (13.2/0.005)	1152	Grouped-RC	**In this work**

Supplementary Information Table 1 | Device performance parameters in reported studies.

Sequence signal	$X_{(-1V)}(t_6)$ - $X_0(t_6)$	$X_{(-3V)}(t_6)$ - $X_0(t_6)$	$X_{(-8V)}(t_6)$ - $X_0(t_6)$	$X_{(-10V)}(t_6)$ - $X_0(t_6)$	$X_{(-15V)}(t_6)$ - $X_0(t_6)$
000001	21.4	50.5	77.1	120.6	353.1
000010	21.8	50.9	77.5	121.1	354.7
000011	25.5	55.5	82.4	127.2	371.2
000011	27.6	58.1	85.2	130.7	380.6
000100	23.9	53.5	80.3	124.6	364.1
000101	28.6	59.4	86.6	132.5	385.3
000110	29.7	60.7	88.0	134.2	390.0
000111	33.4	65.3	92.9	140.3	406.5
001000	24.4	54.2	81.0	125.5	366.5
001001	34.6	66.7	94.4	142.2	411.6
001010	35.2	67.5	95.2	143.3	414.5
001011	35.8	68.2	96.0	144.2	417.0
001100	34.1	66.1	93.8	141.4	409.5
001101	39.0	72.2	100.3	149.5	431.4
001110	74.7	116.5	147.4	208.5	590.6
001111	40.6	74.2	102.4	152.2	438.5
010000	32.7	64.4	92.0	139.2	403.4
010001	34.6	66.8	94.5	142.3	411.9
010010	55.4	92.5	121.9	176.6	504.6
010011	36.5	69.1	96.9	145.4	420.1
010100	54.0	90.8	120.1	174.3	498.2
010101	77.6	120.0	151.3	213.3	603.6
010110	61.6	100.2	130.2	186.9	532.3
010111	37.6	70.5	98.4	147.2	425.0
011000	72.5	113.6	144.5	204.8	580.5
011001	68.8	109.1	139.6	198.7	564.1
011010	39.4	72.7	100.8	150.2	433.3
011011	50.8	86.9	115.9	169.1	484.1
011100	47.6	82.9	111.7	163.8	470.0
011101	41.6	75.5	103.8	153.9	443.2
011110	45.5	80.3	108.8	160.3	460.3
011111	26.8	57.2	84.2	129.5	377.3

Supplementary Information Table 2a | The difference between the reservoir state in each dimension $X_{(Vgs)}$ (t_6) and the initial reservoir state with zero gate bias X_0 (t_6). After the device is input with 32 sequence signals (from ‘000000’ to ‘011111’), the difference between the reservoir state $X_{(Vgs)}$ (t_6) with gate bias and the X_0 (t_6) without gate bias.

Sequence signal	$X_{(-1V)}(t_6)$ - $X_0(t_6)$	$X_{(-3V)}(t_6)$ - $X_0(t_6)$	$X_{(-8V)}(t_6)$ - $X_0(t_6)$	$X_{(-10V)}(t_6)$ - $X_0(t_6)$	$X_{(-15V)}(t_6)$ - $X_0(t_6)$
100000	31.0	62.3	89.7	136.3	395.6
100001	81.2	124.4	156.0	219.2	619.3
100010	78.3	120.9	152.2	214.4	606.6
100011	42.2	76.1	104.4	154.8	445.5
100100	75.5	117.4	148.5	209.8	594.2
100101	53.2	89.8	119.0	173.0	494.7
100110	54.5	91.4	120.8	175.2	500.6
100111	57.7	95.3	124.9	180.4	514.7
101000	49.8	85.6	114.6	167.4	479.6
101001	69.8	110.4	141.0	200.4	568.8
101010	43.0	77.2	105.6	156.2	449.3
101011	89.4	134.6	166.8	232.7	655.8
101100	74.0	115.6	146.5	207.4	587.6
101101	83.0	126.7	158.4	222.2	627.6
101110	87.8	132.6	164.7	230.0	648.7
101111	44.5	79.0	107.5	158.6	455.9
110000	47.0	82.1	110.8	162.7	466.9
110001	56.6	94.0	123.5	178.6	510.0
110010	38.2	71.2	99.2	148.2	427.9
110011	64.5	103.8	134.0	191.7	545.2
110100	71.6	112.5	143.3	203.3	576.5
110101	44.1	78.5	107.0	157.9	454.0
110110	59.3	97.3	127.0	183.0	521.7
110111	60.3	98.6	128.4	184.7	526.4
111000	49.2	84.9	113.8	166.4	477.0
111001	67.7	107.8	138.2	196.9	559.4
111010	63.0	101.9	131.9	189.1	538.2
111011	52.4	88.8	118.0	171.7	491.1
111100	66.3	106.0	136.3	194.6	553.0
111101	85.1	129.3	161.2	225.7	637.0
111110	79.4	122.3	153.7	216.3	611.6
111111	93.6	139.8	172.3	239.6	674.6

Supplementary Information Table 2b | The difference between the reservoir state in each dimension $X_{(Vgs)}$ (t_6) and the initial reservoir state with zero gate bias X_0 (t_6). After the device is input with 32 sequence signals (from '100000' to '111111'), the difference between the reservoir state $X_{(Vgs)}$ (t_6) with gate bias and the X_0 (t_6) without gate bias.

Sequence signal	$\overline{\Delta X_{(310nm)(Vgs)}(t_6)}$	$\overline{\Delta X_{(425nm)(Vgs)}(t_6)}$	$\overline{\Delta X_{(525nm)(Vgs)}(t_6)}$	$\overline{\Delta X_{(650nm)(Vgs)}(t_6)}$	$\overline{\Delta X_{(808nm)(Vgs)}(t_6)}$
000001	118.8	101.0	73.6	50.2	24.9
000010	119.3	101.4	73.9	50.4	25.0
000011	124.0	105.4	76.8	52.4	26.0
000011	126.6	107.6	78.5	53.5	26.6
000100	121.9	103.6	75.6	51.6	25.6
000101	128.0	108.8	79.3	54.1	26.8
000110	129.3	109.9	80.2	54.7	27.1
000111	134.0	113.9	83.1	56.7	28.1
001000	122.6	104.2	76.0	51.8	25.7
001001	135.5	115.2	84.0	57.3	28.4
001010	136.3	115.9	84.5	57.6	28.6
001011	137.0	116.5	84.9	57.9	28.7
001100	134.9	114.7	83.6	57.0	28.3
001101	141.1	120.0	87.5	59.7	29.6
001110	186.7	158.7	115.7	78.9	39.2
001111	143.2	121.7	88.7	60.5	30.0
010000	133.1	113.2	82.5	56.3	27.9
010001	135.6	115.2	84.0	57.3	28.4
010010	162.0	137.7	100.4	68.5	34.0
010011	137.9	117.2	85.5	58.3	28.9
010100	160.2	136.2	99.3	67.7	33.6
010101	190.4	161.8	118.0	80.5	39.9
010110	170.0	144.5	105.4	71.9	35.7
010111	139.3	118.4	86.4	58.9	29.2
011000	183.8	156.2	113.9	77.7	38.6
011001	179.1	152.2	111.0	75.7	37.6
011010	141.7	120.4	87.8	59.9	29.7
011011	156.2	132.8	96.8	66.0	32.8
011100	152.2	129.3	94.3	64.3	31.9
011101	144.5	122.8	89.6	61.1	30.3
011110	149.4	127.0	92.6	63.2	31.3
011111	125.7	106.8	77.9	53.1	26.4

Supplementary Information Table 3a | The $X_{(\lambda)(Vgs)}(t_6)$ of VOFET-DR with different bias V_{GS} when different wavelength sequence light pulses are input. After inputting 32 light sequence pulse signals with different wavelengths (000000 to 011111) to VOFET-DR with different gate biases V_{GS}, the sample standard deviation of $X_{(\lambda)(Vgs)}(t_6)$ is obtained. For example, the $\overline{\Delta X_{(310nm)(Vgs)}(t_6)}$ of the sequence signal 000001 is obtained by calculating the standard deviation between $X_{(310nm)(-1V)}(t_6)$, $X_{(310nm)(-3V)}(t_6)$, $X_{(310nm)(-8V)}(t_6)$, $X_{(310nm)(-12V)}(t_6)$, and $X_{(310nm)(-15V)}(t_6)$.

Sequence signal	$\overline{\Delta X_{(310nm)(Vgs)}(t_6)}$	$\overline{\Delta X_{(425nm)(Vgs)}(t_6)}$	$\overline{\Delta X_{(525nm)(Vgs)}(t_6)}$	$\overline{\Delta X_{(650nm)(Vgs)}(t_6)}$	$\overline{\Delta X_{(808nm)(Vgs)}(t_6)}$
100000	130.9	111.3	81.2	55.4	27.5
100001	194.9	165.6	120.8	82.4	40.9
100010	191.2	162.5	118.6	80.9	40.1
100011	145.2	123.4	90.0	61.4	30.4
100100	187.7	159.5	116.3	79.4	39.4
100101	159.2	135.3	98.7	67.3	33.4
100110	160.9	136.8	99.7	68.0	33.7
100111	164.9	140.2	102.2	69.7	34.6
101000	154.9	131.7	96.0	65.5	32.5
101001	180.4	153.3	111.8	76.3	37.8
101010	146.2	124.3	90.7	61.8	30.7
101011	205.3	174.5	127.3	86.8	43.1
101100	185.8	157.9	115.2	78.6	39.0
101101	197.2	167.6	122.3	83.4	41.4
101110	203.3	172.8	126.0	86.0	42.6
101111	148.1	125.9	91.8	62.6	31.1
110000	151.3	128.6	93.8	64.0	31.7
110001	163.6	139.0	101.4	69.2	34.3
110010	140.1	119.1	86.9	59.2	29.4
110011	173.7	147.6	107.7	73.4	36.4
110100	182.6	155.2	113.2	77.2	38.3
110101	147.6	125.4	91.5	62.4	31.0
110110	166.9	141.9	103.5	70.6	35.0
110111	168.3	143.0	104.3	71.2	35.3
111000	154.2	131.0	95.6	65.2	32.3
111001	177.7	151.0	110.2	75.1	37.3
111010	171.7	145.9	106.4	72.6	36.0
111011	158.2	134.5	98.1	66.9	33.2
111100	175.9	149.5	109.5	74.4	36.9
111101	199.9	169.9	123.9	84.5	41.9
111110	192.7	163.7	119.4	81.5	40.4
111111	210.7	179.1	130.6	89.1	44.2

Supplementary Information Table 3b | The $X_{(\lambda)(Vgs)}(t_6)$ of VOFET-DR with different bias V_{GS} when different wavelength sequence light pulses are input.

	X	Y	Relative permittivity
Gate (Si)	500 nm	50 nm	-
Dielectric layer(SiO_2)	500 nm	100 nm	4.5
Passivation layer (PVA)	500nm	20 nm	3.5
Source (MXene)	75 nm	3 nm	-
Active layer	500 nm	100 nm	2.5
Drain (Au)	500 nm	50 nm	-

Supplementary Information Table 4 | COMSOL related simulation parameters.

Supplementary Note 1. Reservoir computing model and physical device design based on delay-feedback system

1. Reservoir computing model

First, as shown in Figure N1.1, the reservoir computing network is a Recurrent Neural Network model based on a delayed feedback system. The core idea is to process the input signal through a fixed, sparse, randomly connected recurrent network (called a reservoir), where the node needs to receive the input signal and its own feedback signal, and then update its state according to a pre-set connection weight and nonlinear activation function. After that, the state of the reservoir is mapped to the output layer by linear output weights, and the timing signal is predicted by a simple algorithm of the output layer. Therefore, the relationship between its input I and output O can be described by the following mathematical equation:

$$x(t+1) = f(W_{res} * x(t) + W_{in} * u(t))$$
$$y(t) = W_{out} * x(t)$$

Where $x(t)$ is the network state at the current moment. $x(t+1)$ is the network state at the next moment. $u(t)$ is the input at the current moment. $y(t)$ is the output at the current moment. W_{res} is the recursive weight matrix of the reservoir, which is used to control the update of the current network state, W_{in} is the input weight matrix, which is used to control the effect of the input signal on the state vector. W_{out} is the output weight matrix, which is used to map the state vector of the reservoir to the output. *f ()* represents a nonlinear function.

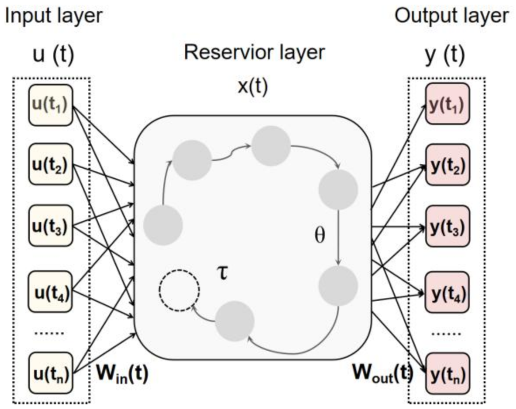

Figure N1.1 Model diagram of reservoir computing network based on delayed feedback system.

Therefore, in a reservoir computing network based on a delayed feedback system, multiple reservoir nodes are required to feed back their own output as input to satisfy the recursive condition, which means that the output of the reservoir node will directly or indirectly affect its own input. So, in physical reservoir computing, this requires the

physical nodes to be able to physically respond, transmit, and memorize signals, expressed as a series of node self-feedback. This can be described by the following equation:

$$\frac{dx(t)}{dt} = f(t, x(t), x(t-\tau))$$

$$\theta = \tau/N$$

Where τ is the duration of the delay and *N* is the number of nodes and the θ is the time-step and *f ()* is a system function that depends on the physical system.

It can be found that in order to satisfy the mathematical architecture of RC at the physical device level, the output states of the physical devices must satisfy: nonlinear response characteristics, short-term memory characteristics and a large number of reservoir states. Among them, the number of reservoir states, as nodes, determines the computational capacity of reservoir computing, and the number depends greatly on the former two.

2. Physical coefficients of the devices

1) For nonlinear response characteristics, as shown in Figure N1.2 and in Figures 2d and 2e of the manuscript, the device generates nonlinear response behavior induced by light pulses or voltage. By fitting its mathematical expression as follows:

$$I = I_{(t-1)} + A\,[1 - exp\frac{(-(t-t_0))}{\tau}]$$

$$A \sim f(F)$$

Where $I_{(t-1)}$ is the initial current state, the A coefficient is the difference between $I_{(t-1)}$ and $I_{(\infty)}$, which belongs to the intrinsic characteristics of the device and depends on the feedback intensity F of the device.

The mathematical equation describes the evolution process of the reservoir state after the physical reservoir is fed a signal, which can be regarded as a reservoir state space. Therefore, it can be found that there is a significant relationship between the number of reservoir state spaces and A coefficient. In order to make the reservoir have as many state spaces as possible to provide as many reservoir states as possible, as shown in the Figure N1.3a, the A coefficient needs to be able to be regulated in a large range.

2) On the other hand, for the short-term memory characteristics, the mathematical expression it is fitted:

$$I(t) = I_{spike} + D_1 exp\left(-{}^{t}/_{\tau_1}\right) + D_2 exp\left(-{}^{t}/_{\tau_2}\right)$$

Where, τ_1 and τ_2 represent the characteristic time of the fast decay and slow decay process, respectively. D_1 and D_2 represent the prefactor. I_{spike} represent the current constant. Since the physical nodes in the reservoir need to satisfy the point-wise separation property of the reservoir, in other words, be able to respond significantly to different sequence signals, we use the characteristic time τ_1 of the device to better evaluate the mapping ability of the device to the sequence signal. Therefore, the decay process of the memory current can be seen as:

$$I(t) = I_{spike} + D_1 exp\left(-{}^{t}/_{\tau}\right)$$

The time characteristic τ determines the decay rate of the memory current, which directly affects the evolution process of the reservoir state, the feedback relationship between the input $I(t-1)$ and output $I(t)$ of the signal in the reservoir state space. In order to make the reservoir have more reservoir state space to provide as many reservoir states as possible, as shown in the Figure N1.3b, the time characteristic τ needs to be adjustable on a large scale.

Therefore, based on the above analysis, we need to meet the large-scale feedback intensity F and time characteristics τ for the design of the device to provide as many reservoir states as possible.

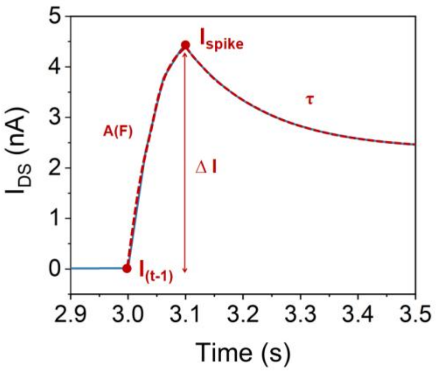

Figure N1.2 Nonlinear function extracted from the light response curve (I-t) of the device.

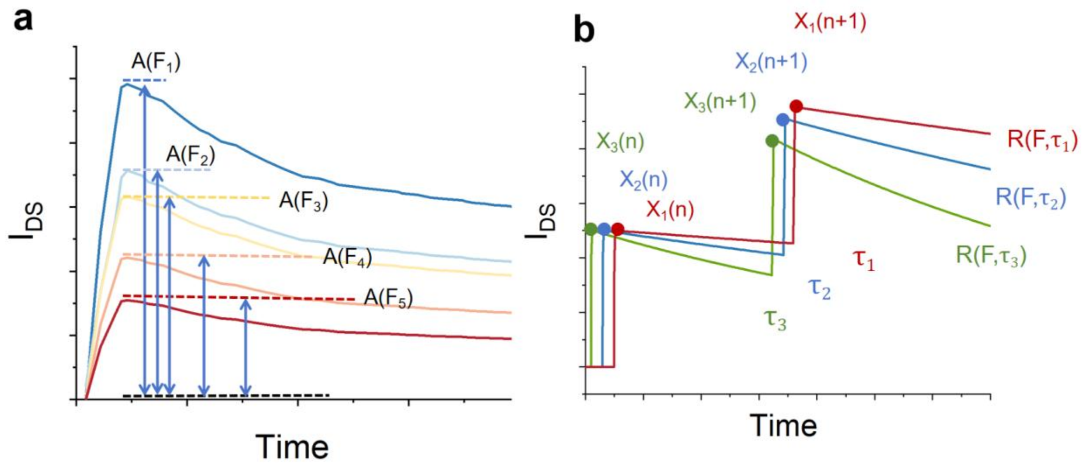

Figure N1.3 **a** Different reservoir states due to different feedback intensities. **b** Different reservoir states due to different temporal characteristics.

3. Design strategies for physical reservoir devices

Since the dynamic range of A coefficient is related to the feedback intensity F of the device, the problem of the dynamic range of A coefficient can be solved by increasing F. In the process of current rise, the relationship between the change amount of photocurrent ΔI and the input light intensity can be regarded as macroscopically:

$$\frac{\Delta I}{P_{in}} = \frac{I_{spike} - I_0}{P_{in}} = F$$

Where scaling factor *F*, the feedback strength, shows the weight between the output and the input of the device.

Currently, optoelectronic devices used in reservoir computing mainly focus on dynamic optoelectronic memory resistors. However, due to the limited ports, it is difficult to further adjust the physical mechanism of the device or the carrier transmission process, resulting in a relatively fixed dynamic range of the feedback intensity F. As a result, the output of the device is almost determined by the most recent input, making the device itself a relatively fixed nonlinear transformation function, thereby limiting its ability to provide a more diverse reservoir state space to the system.

After the introduction of field effect characteristics, the gate voltage at the third terminal can more accurately regulate the distribution of carriers in the photoelectric field effect transistor, thus realizing a richer carrier transport process. Therefore, using field effect transistors to design reservoirs is greatly beneficial to improve the dynamic range of the feedback intensity F to create more reservoir states. However, for traditional transistors, the carriers in the channel are transported in the horizontal direction. The micro-level long transport distance results in severe charge loss of the carriers due to interface defects, bulk defects, and so on during the transport process.

This greatly limits the carrier transport efficiency and gate control ability, thereby affecting the feedback intensity of the device.

And for the new vertical architecture transistor, it not only has the basic function of gate electric field modulation, but also due to its source and drain being located at the bottom and top of the semiconductor layer respectively, carriers are influenced by the electric field for transport in the vertical direction. This means that the transport distance is only determined by the thickness of the active layer (which is ~65 nm in this work). This nano-level channel length significantly reduces the carrier transport distance and reduces charge loss during the transport process, thereby improving carrier transport efficiency. In addition, the field distribution between the source and drain electrodes under gate control is non-uniform. The region near the source electrode will experience stronger gate control. The non-uniform distribution of the adjustable electric field is beneficial for regulating the separation balance between holes and electrons inside photoelectron devices, reducing the recombination caused by different electron-hole transmission rates, and enhancing the gate control capability of the device. As a result, the dynamic range of the feedback intensity F can be effectively improved.

On the other hand, the short-term memory characteristics of the device are essentially determined by the charge trapping and de-trapping processes in the channel. The energy state of the trapped charge greatly affects the process of de-trapping, resulting in time characteristics τ at different scales. For the mechanism of charge trapping, there is a functional relationship between the time characteristic τ and the charge activation energy E_a and the ambient temperature T, which can be described by the following physical equations:

$$\tau = \tau_0 \, exp\,(\frac{E_a}{k_B T})$$

In optoelectronic devices, photogenerated charges are generated by the photoelectric effect, and photons of different energies can excite photogenerated electron-hole pairs at different energy levels. Photogenerated electron-hole pairs at different energy levels have different activation energies, which is the minimum energy required to form a photogenerated charge. Therefore, under room temperature conditions, we choose an organic semiconductor (P0FDIID) with broad-spectrum absorption characteristics as the channel material for the device, with an absorption spectrum ranging from 300 nm to 1000 nm. By exciting photo-generated electrons in different energy states through photons with different frequencies, we can provide varying activation energies, as shown in the Figure N1.4. Next, by introducing an electron acceptor organic semiconductor (N2200) with energy levels that match, a PN heterojunction system is formed. The potential barrier generated at the interface can trap and de-trap photo-generated electrons with different activation energies during the transport process, resulting in a diverse time characteristic.

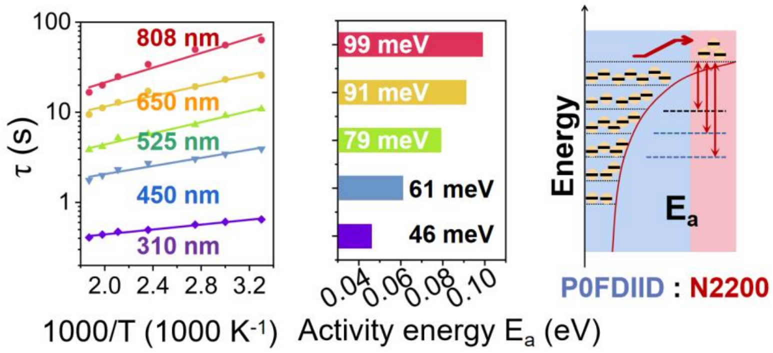

Figure N1.4 Charge activation energy generated by light pulses of different wavelengths.

Supplementary Note 2. The extraction of nonlinear temporal characteristics τ

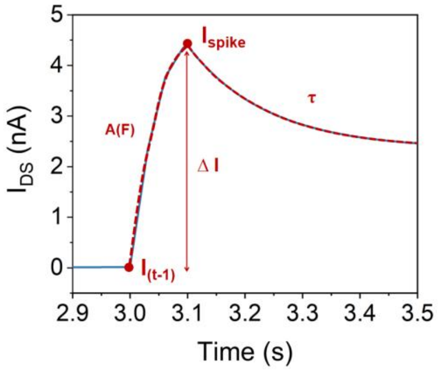

When the input light pulse ends, the output current of the reservoir physical node exhibits the characteristic of exponential decay. It can be observed that the decay of the current has two regimes: a rapid decay immediately after the light pulse ends, followed by a slower decay after a few milliseconds. Therefore, this result can be modeled using a double exponential function:

$$I(t) = I_{spike} + D_1 exp\left(-{}^{t}/_{\tau_1}\right) + D_2 exp\left(-{}^{t}/_{\tau_2}\right) \quad (1)$$

Where, τ_1 and τ_2 represent the characteristic time of the fast decay and slow decay process, respectively. D_1 and D_2 represent the pre-factor. I_{spike} represent the current constant. Since the physical nodes in the reservoir need to satisfy the point-wise separation property of the reservoir, in other words, be able to respond significantly to different sequence signals, we use the characteristic time τ_1 of the device to better evaluate the mapping ability of the device to the sequence signal.

Supplementary Note 3. The method of computing standard deviation

In statistics, standard deviations are used to assess the degree of data dispersion. In reservoir computing, physical nodes with rich reservoir states are needed to efficiently map the nonlinear time characteristics of sequential signals into the reservoir space. Since the temporal characteristics of VOFET-DR depend on the wavelength of the input light pulse and the gate voltage bias, the same sequence signal can be mapped to different reservoir spaces. Therefore, the dispersion of different reservoir states based on the same sequence signals can be evaluated using the standard deviation to measure the richness of reservoir states.

The formula for calculating the standard deviation is as follows:

$$SD = \sqrt{\frac{1}{N}\sum_{i=1}^{N}(X_i - \mu)^2} \quad (2)$$

where, X_i represents the series of data to be evaluated. μ is the mean of the series of data. N represents the number of series data.

Supplementary Note 4. The method of computing activation energy

For charge trapping, the decay constant τ is functionally related to the activation energy E_a and the ambient temperature T. It can be described by the following equation:

$$\tau = \tau_0 \, exp\,(\frac{E_a}{k_B T})$$

where τ_0 and k_B represent the thermal constant and the Boltzmann constant, respectively. E_a is typical the activation energy for charge trapping. According to the Arrhenius equation, the activation energy is inversely proportional to the ambient temperature T. As the ambient temperature increases, the activation energy will decrease. Conversely, as the ambient temperature decreases, the activation energy will increase. This is because high temperatures increase the average kinetic energy of molecules, making it easier to overcome the energy barrier between reactants, thereby reducing the activation energy. Low temperatures slow down the movement of molecules, making it more difficult to overcome the energy barrier, resulting in increased activation energy. Therefore, changes in temperature can trigger changes in the decay constant τ, as shown in Figure N4. Since the value of τ can be directly obtained from the decay of I_{DS}, the corresponding activation energy E_a can be calculated by testing the I_{DS} induced by light pulses of specific wavelengths at different ambient temperatures.

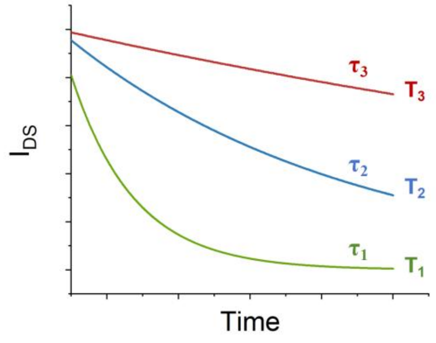

Figure N4. Current decay varies with ambient temperature.

Supplementary Note 5. The effect of different mixing ratios.

We investigated the effect of different mixing ratios on the memory current of the device, as shown in Figure N5. Based on different p/n semiconductors blending ratios, BHJ is used as the transistor channel, and the device is subjected to the same optical pulse. As shown in Figure N5a, at a low n-type blending ratio, the device exhibits better memory retention characteristics. However, as the n-type blending ratio increases, as shown in Figures N5b and N5c, the memory effect of the device gradually decreases, transitioning from long-term memory to short-term memory. For reservoir computing, devices serving as physical nodes in the reservoir layer need to possess short-term memory characteristics to meet the recursive requirements. If long-term memory characteristics are used, although the recursive condition can be satisfied, it is difficult to map the input signal to high-dimensional space due to its lack of nonlinearity. Therefore, after experimental optimization, a blending ratio of 20% wt (N2200) is adopted in this work.

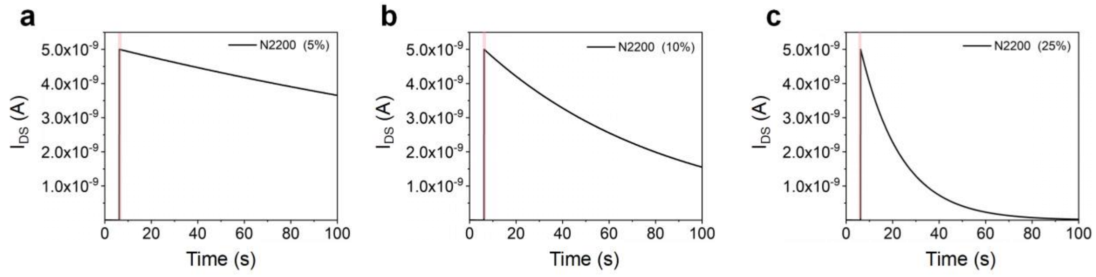

Figure N5 The impact of different blending ratios on the memory characteristics induced by optical pulses in the device (450nm, pulse width Δt = 100 ms, V_{DS} = -1 V, light intensity P_{in} = 0.01mW/cm^2). a The blending ratio of N2200 is 5% wt. **b** The blending ratio of N2200 is 10% wt. **c** The blending ratio of N2200 is 25% wt.

Reference

[1] Du, C., *et al.* Reservoir computing using dynamic memristors for temporal information processing. *Nat. Commun.* **8**, 2204 (2017).

[2] Zhong, Y., *et al.* Dynamic memristor-based reservoir computing for high-efficiency temporal signal processing. *Nat. Commun.* **12**, 408 (2021).

[3] Zhang, Z., *et al.* In-sensor reservoir computing system for latent fingerprint recognition with deep ultraviolet photo-synapses and memristor array. *Nat. Commun.* **13**, 6590 (2022).

[4] Liu, K., *et al.* An optoelectronic synapse based on α-In_2Se_3 with controllable temporal dynamics for multimode and multiscale reservoir computing. *Nat. Electron.* **5**, 761 – 773 (2022).

[5] Chen, Z., Li, W., Fan, Z. *et al.* All-ferroelectric implementation of reservoir computing. *Nat. Commun.* **14**, 3585 (2023).

[6] Tan, H., *et al.* Dynamic machine vision with retinomorphic photomemristor-reservoir computing. *Nat. Commun.* **14**, 2169 (2023).

[7] Wu, X., *et al.* Wearable in-sensor reservoir computing using optoelectronic polymers with through-space charge-transport characteristics for multi-task learning. *Nat. Commun.* **14**, 468 (2023).
